# Diagnostic Value of Fractional Shortening and E-Point Septal Separation in Predicting Left Ventricular Longitudinal Strain in Dyspneic Emergency Patients

**DOI:** 10.3390/medicina62020258

**Published:** 2026-01-26

**Authors:** Mustafa Ucar, Muhammed Ikbal Sasmaz, Doguhan Bitlisli, Akkan Avci

**Affiliations:** 1 Department of Cardiology, Faculty of Medicine, Celal Bayar University, 45030 Manisa, Türkiye; mustafa.ucar@cbu.edu.tr; 2 Department of Emergency Medicine, Faculty of Medicine, Celal Bayar University, 45030 Manisa, Türkiye; dr.doguhan@gmail.com; 3 Department of Emergency Medicine, Adana City Research and Training Hospital, Health Science University, 01060 Adana, Türkiye; drakkanavci@gmail.com

**Keywords:** dyspnea, point-of-care ultrasound, e-point septal separation, fractional shortening, global longitudinal strain, ejection fraction, emergency department

## Abstract

*Background and Objectives:* Dyspnea is a common chief complaint in the emergency department. While global longitudinal strain and biplane ejection fraction are reliable markers of left ventricular systolic function, their assessment requires advanced echocardiographic tools and expertise. Simple point-of-care ultrasound parameters, such as E-point septal separation and fractional shortening may serve as practical alternatives for rapid bedside evaluation. *Materials and Methods:* EPSS and FS were measured by emergency physicians using POCUS, while reference EF and GLS were obtained by cardiologists via transthoracic echocardiography. Correlation analyses, receiver operating characteristic curves, and agreement statistics were used to evaluate the diagnostic accuracy of EPSS and FS for predicting reduced EF (<50%) and GLS (<16%). *Results:* Reduced EF was present in 54.0% and reduced GLS in 55.6% of patients. EPSS showed strong negative correlations with EF (ρ = −0.834) and GLS (ρ = −0.782), while FS correlated positively with EF (ρ = 0.773) and GLS (ρ = 0.714), all *p* < 0.001. ROC analysis demonstrated excellent diagnostic accuracy of EPSS (AUC = 0.922 for EF; 0.949 for GLS) and good accuracy of FS (AUC = 0.874 for EF; 0.865 for GLS). Optimal cut-off values were EPSS ≥ 7.0 mm and FS ≤ 25%. Agreement with reference TTE was good for EPSS (κ = 0.676 for EF; κ = 0.738 for GLS) and moderate for FS (κ ≈ 0.56). *Conclusions:* Both EPSS and FS measured by POCUS provide reliable estimates of left ventricular systolic function in dyspneic ED patients, with EPSS demonstrating superior diagnostic performance.

## 1. Introduction

Dyspnea is defined by the American Thoracic Society as a subjective experience of breathing discomfort that comprises qualitatively distinct sensations of varying intensity [1]. Although its prevalence may vary regionally, dyspnea accounts for approximately 5–10% of emergency department (ED) visits [2,3]. The severity and perception of dyspnea are highly variable and are influenced by the underlying pathology, sociocultural factors, and regional differences. While the most effective treatment approach involves identifying and managing the underlying cause, establishing an accurate diagnosis in dyspneic patients remains a clinical challenge. The growing elderly population and the increasing diversity and overlap of comorbidities further complicate diagnostic efforts, often leading to delayed or even missed diagnoses [4].

Heart failure (HF), pneumonia, and exacerbations of chronic obstructive pulmonary disease (COPD) are among the most common causes of dyspnea. However, many other etiologies may be encountered, with dyspnea-related mortality reaching up to 8% in some reports [4,5]. Given its association with increased risk of mortality, hospitalization, intensive care unit admission, and repeated ED visits, dyspnea is a critical predictor of poor clinical outcomes. Cardiovascular conditions, particularly HF, are among the leading causes of dyspnea and are frequently encountered in the ED setting [2]. Moreover, the severity of dyspnea is generally greater in patients with cardiovascular etiologies and is correlated with mortality risk. Therefore, the diagnosis of HF is essential to facilitate early intervention and improve patient outcomes [6].

The clinical presentation of acute HF can vary widely, ranging from mild symptom exacerbation to cardiogenic shock and acute pulmonary edema. In 14–29% of cases, a definitive diagnosis cannot be made due to advanced patient age, presence of multiple comorbidities, and limitations in accessing specialized diagnostic tools [7]. HF is a complex clinical syndrome with a multifactorial pathophysiology, often requiring a multimodal diagnostic approach. Despite its complexity, HF is primarily classified based on left ventricular ejection fraction (LVEF) and clinical staging [8].

Transthoracic echocardiography (TTE) remains the cornerstone imaging modality for evaluating cardiac structure and function in routine clinical practice. However, comprehensive echocardiographic assessment requires extensive training, and the availability of skilled personnel in emergency settings may be limited. In response to these limitations, cardiac point-of-care ultrasound (POCUS) has emerged as a valuable bedside tool for rapidly assessing cardiac, pulmonary, and vascular structures. POCUS provides both qualitative and quantitative data, offering a practical solution in the ED setting [9].

Among POCUS-derived parameters, E-point septal separation (EPSS) is a simple and rapid M-mode measurement defined as the minimal distance between the anterior mitral valve leaflet and the interventricular septum during early diastole (EPSS > 7 mm indicates reduced LVEF < 50%) [10]. EPSS is obtained easily with minimal training and demonstrates a strong inverse correlation with LVEF, allowing rapid assessment of systolic dysfunction—especially valuable in emergency contexts [11,12].

Fractional shortening (FS) is an M-mode-based echocardiographic parameter frequently used in the ED. Although it does not directly measure LVEF, FS demonstrates a strong correlation with it. FS is calculated using the formula: FS (%) = (LVIDd − LVIDs)/LVIDd × 100, where LVIDd and LVIDs represent the left ventricular internal diameters in diastole and systole, respectively. Normal FS values range between 25–45%, corresponding to an approximate LVEF of 55–70% [13,14].

Myocardial strain refers to the deformation of the myocardium during the cardiac cycle. In the left ventricle, strain can be measured in longitudinal, circumferential, and radial planes [15]. Global longitudinal strain (GLS), evaluated by speckle-tracking echocardiography, has become the most widely used strain parameter. GLS measures the longitudinal deformation of myocardial speckles and is considered less dependent on geometric assumptions, preload, and afterload than LVEF [16]. A meta-analysis involving 24 studies identified normal GLS values ranging from 15.9% to 21.1% [17]. Compared to LVEF, GLS has been shown to be a stronger predictor of all-cause and cardiovascular mortality, hospitalization, and arrhythmic events [18].

In this study, we aimed to investigate the correlation between FS, EPSS, and GLS in patients presenting to the emergency department with dyspnea, with the objective of evaluating FS and EPSS as practical and accessible tools for assessing left ventricular systolic function in resource-limited settings.

## 2. Materials and Methods

### 2.1. Study Design

This was a single-center, prospective observational study conducted at the Emergency Department of Manisa Celal Bayar University Faculty of Medicine Hospital between January 2024 and December 2025. Patients presenting to the Emergency Department with dyspnea symptoms who underwent initial POCUS for differential diagnosis were included. Cardiac ultrasound examinations performed for POCUS were conducted by an emergency medicine specialist. The equipment used was a Mindray DC-40 Ultrasound System with a P4-2 phased-array transducer (Mindray Corp., Shenzhen, China, 2022). FS and EPSS values were recorded on study forms.

Subsequently, all patients underwent a comprehensive echocardiographic examination performed independently by a cardiologist, regardless of the initial findings. Echocardiography was performed using the Philips Affiniti 50G with a S4-2 (Operating frequency range from 4 to 2 MHz) transducer. LVEF was calculated using the biplane Simpson method, and GLS Global longitudinal strain (GLS) was assessed using vendor-specific speckle-tracking software (Philips QLAB, Philips Medical Systems, Andover, MA, USA).

Patients were excluded if they were hemodynamically unstable, required resuscitation, had electrocardiographic evidence of significant ST-segment elevation or atrial fibrillation, had a history of valve surgery or severe valvular disease, were under the age of 18 years, had inadequate echocardiographic windows, or declined to participate. In addition, patients who presented with a clear exacerbation of obstructive lung disease based on history and physical examination were excluded from the study.

The study was approved by the Ethics Committee of Celal Bayar University (decision number: 493, approval date: 19 June 2023) and written informed consent was obtained from patients or their immediate relatives.

### 2.2. Data Collection and Clinical Assessment

Demographic data (age, sex), comorbidities, vital signs, echocardiographic parameters, final diagnoses, and clinical outcomes were recorded. Standard laboratory and radiological investigations were performed in all patients according to clinical indications.

### 2.3. Echocardiographic Measurements

**Figure 1:**
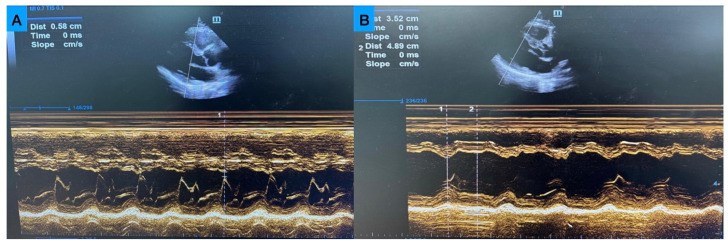
Measurement of E-point septal separation (EPSS) and fractional shortening (FS) using point-of-care ultrasound (POCUS). (**A**) EPSS was measured in early diastole on parasternal long-axis (PLAX) M-mode POCUS as the distance between the tip of the anterior mitral valve leaflet and the interventricular septum. (**B**) FS was calculated from M-mode images obtained at the mid-ventricular level in PLAX, with the ultrasound beam positioned perpendicular to the interventricular septum and posterior wall. Left ventricular internal diameters at end-diastole (LVIDd) and end-systole (LVIDs) were measured, and FS was derived as FS = (LVIDd − LVIDs)/LVIDd × 100.

EPSS was measured in early diastole on parasternal long-axis (PLAX) M-mode echocardiography as the distance between the anterior mitral valve leaflet tip and the interventricular septum. FS was calculated from M-mode images obtained at the mid-ventricular level in PLAX, with the ultrasound beam perpendicular to the interventricular septum and posterior wall (Figure 1).

**Figure 2:**
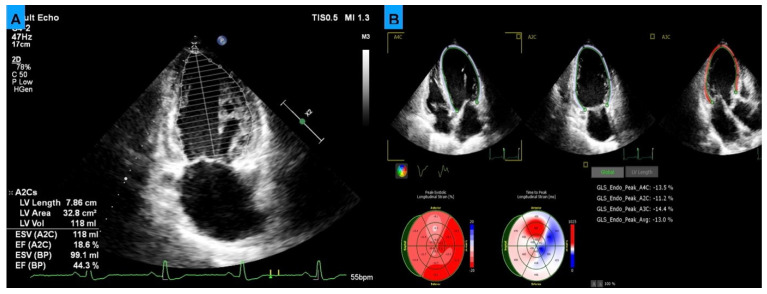
Reference transthoracic echocardiography (TTE) parameters. (**A**) Left ventricular ejection fraction (EF) calculated using the biplane Simpson’s method from apical four-chamber and two-chamber views. (**B**) Global longitudinal strain (GLS) obtained by speckle-tracking echocardiography, showing strain curves and a bull’s-eye plot for quantitative assessment of left ventricular systolic function.

As the reference standard, all patients underwent transthoracic echocardiography (TTE) performed by a cardiologist. LVEF was measured using the biplane Simpson method, and GLS was assessed using speckle-tracking echocardiography (Figure 2).

Continuous variables included FS, EPSS, LVEF, and GLS values. For categorical analyses, thresholds commonly reported in the literature were applied:

- EPSS: >7 mm = “reduced”, ≤7 mm = “normal” [11,12];
- FS: <25% = “reduced”, ≥25% = “normal” [13,14];
- EF: <50% = “reduced”, ≥50% = “normal” [8];
- GLS: <16% = “reduced”, ≥16% = “normal” [19].

The primary endpoint was to evaluate the diagnostic accuracy of EPSS and FS obtained by emergency POCUS in predicting reduced LVEF and GLS, using cardiologist-performed echocardiography as the reference standard.

### 2.4. Statistical Analysis

All data were analyzed using SPSS software (version 27, IBM Corp., Armonk, NY, USA). Continuous variables were expressed as mean ± standard deviation (SD), median with interquartile range (IQR), or percentages, as appropriate. The distribution of continuous variables was assessed using the Kolmogorov–Smirnov and Shapiro–Wilk tests as well as histogram inspection. LVEF and GLS were analyzed both as continuous variables and as categorical variables based on the predefined cut-off values (<50% for LVEF, <16% for GLS).

The diagnostic accuracy of EPSS and FS in predicting reduced LVEF and GLS was evaluated using receiver operating characteristic (ROC) curve analysis, reporting sensitivity, specificity, and area under the curve (AUC) values. Optimal cut-off points were determined using the Youden Index. For correlation analyses, Spearman’s rank correlation was applied as LVEF and EPSS did not follow a normal distribution. Agreement between categorical FS and EPSS classifications and LVEF/GLS categories was assessed using Cohen’s kappa statistic. A *p*-value < 0.05 was considered statistically significant.

## 3. Results

### 3.1. Demographic and Baseline Characteristics

A total of 63 patients were included in the study. The mean age was 61.7 ± 14.2 years (median 64; range 21–85 years), and 60.3% (*n* = 38) were male. At least one comorbidity was identified in 88.9% of patients (*n* = 56), with hypertension (54.0%, *n* = 34) and coronary artery disease (39.7%, *n* = 25) being the most prevalent conditions. Baseline demographics, vital signs, and comorbidities are summarized in Table 1.

### 3.2. Final Diagnoses and Emergency Department Outcomes

The most common final diagnoses were acute coronary syndrome (34.9%), decompensated heart failure (27.0%), and pneumonia (12.7%). Regarding emergency department outcomes, 54.0% of patients were admitted to the intensive care unit, 14.3% to the ward, and 31.7% were discharged. Details are shown in Table 2.

### 3.3. Echocardiographic Parameters

Valid cardiac measurements were obtained in all 63 patients. The mean global longitudinal strain (GLS) was 14.28 ± 3.87%. The mean biplane ejection fraction (EF) was 46.58 ± 11.81%. The mean fractional shortening (FS) was 26.54 ± 8.18%. The mean EPSS value was 8.7 ± 3.5 mm. These results are presented in Table 3.

### 3.4. ROC Analysis for EPSS and FS

EPSS and FS demonstrated high diagnostic accuracy in detecting reduced EF. The AUC for EPSS was 0.922, and for FS it was 0.874. The optimal thresholds determined by the Youden index were ≥7.05 mm for EPSS (sensitivity 0.861, specificity 0.815) and ≤28.15% for FS (sensitivity 0.778, specificity 0.815).

**Figure 3:**
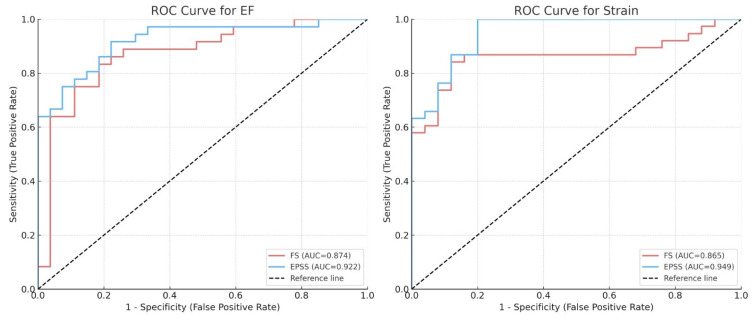
Receiver operating characteristic (ROC) curves of EPSS and FS for predicting reduced ejection fraction (EF) and global longitudinal strain (GLS). AUC: area under the curve; EF: ejection fraction; FS: fractional shortening; EPSS: E-point septal separation; GLS: global longitudinal strain.

Similarly, both parameters showed high discriminatory performance for reduced GLS. EPSS had an AUC of 0.949, while FS had an AUC of 0.865. The optimal thresholds were ≥7.05 mm for EPSS (sensitivity 0.868, specificity 0.880) and ≤24.95% for FS (sensitivity 0.684, specificity 0.920). A summary of the ROC analysis is provided in Table 4, and the ROC curves are depicted (Figure 3).

### 3.5. Correlation Analysis

EPSS measured in the emergency setting showed strong negative correlations with biplane EF (ρ = −0.834), FS (ρ = −0.784), and GLS (ρ = −0.782) (all *p* < 0.001). Reference echocardiographic parameters were also strongly interrelated: EF and GLS (ρ = 0.866), EF and FS (ρ = 0.773), and FS and GLS (ρ = 0.714), all *p* < 0.001. These correlations are summarized in Table 5.

### 3.6. Agreement Analysis (Kappa Statistics)

When categorized according to widely accepted cut-off values, EPSS ≥ 7.0 mm demonstrated good agreement with both reduced EF (κ = 0.676) and reduced GLS (κ = 0.738).

In contrast, FS ≤ 25% demonstrated moderate agreement with both reduced EF (κ = 0.563) and reduced GLS (κ = 0.566). Corresponding 2 × 2 distributions and diagnostic measures are presented in Table 6.

Overall, EPSS demonstrated superior diagnostic accuracy compared with FS in predicting both reduced EF and reduced GLS in the emergency setting.

## 4. Discussion

In this single-center study, we demonstrated that both EPSS and FS, when measured in the ED, exhibited strong correlations with biplane EF and GLS. Importantly, EPSS and FS showed high diagnostic performance for predicting reduced EF and GLS, with EPSS ≥ 7.0 mm and FS ≤ 25% emerging as optimal cut-off values. These findings suggest that simple echocardiographic parameters, which can be rapidly and reliably obtained in the emergency setting, may serve as practical alternatives to advanced imaging modalities in the initial assessment of dyspneic patients.

Previous studies have emphasized the role of GLS as a sensitive marker of left ventricular systolic function, often detecting subtle dysfunction even when EF appears preserved [13,15,16]. However, GLS assessment requires advanced software and expertise, which limits its availability in time-sensitive emergency scenarios [17,18]. Our results show that EPSS and FS, which can be derived from standard two-dimensional echocardiography without additional equipment, correlate strongly with GLS (ρ = −0.782 and ρ = 0.714, respectively). These observations are consistent with earlier reports demonstrating the utility of EPSS and FS in reflecting left ventricular contractile performance [11,12,14], while highlighting their potential role as surrogate markers of GLS in acute care practice.

EPSS has been consistently reported as a robust marker of systolic dysfunction. Emergency ultrasound studies have shown that EPSS >7 mm is strongly associated with reduced EF, and even novice sonographers could reliably obtain this measurement in ED settings [10,11,12]. Our findings confirm these reports, demonstrating a strong negative correlation between EPSS and both EF and GLS. The observed diagnostic accuracy (AUC = 0.949 for GLS) further supports EPSS as a powerful predictor of impaired longitudinal myocardial function, often more sensitive than EF in detecting subtle dysfunction [15,16,17,19].

Fractional shortening, a long-established index of left ventricular systolic performance, has also been validated against EF in multiple clinical contexts [13,14,20]. In our study, FS showed moderate-to-strong correlations with EF and GLS, with acceptable diagnostic accuracy for reduced systolic function. However, compared with EPSS, FS was less predictive, particularly for GLS. This limitation aligns with prior observations that FS is influenced by ventricular geometry and loading conditions, which may reduce its sensitivity in detecting subtle impairments in longitudinal myocardial mechanics [16]. Nonetheless, FS remains a simple and valuable bedside index that can complement EPSS in emergency practice.

To our knowledge, this is among the first studies to systematically compare EPSS and FS against GLS in dyspneic patients presenting to the ED. The stronger alignment of EPSS with GLS suggests that it may be a valuable surrogate for advanced strain imaging, especially in resource-limited or time-sensitive scenarios. FS, while somewhat less reliable, may still provide clinically useful information, particularly when EPSS measurement is not feasible. These results highlight the potential clinical implications of incorporating EPSS and FS into routine ED evaluation, enabling timely decision-making and triage of patients with suspected heart failure or other causes of dyspnea [7,8,9,21,22,23].

Taken together, our results suggest that incorporating EPSS into emergency echocardiographic assessment could improve early identification of left ventricular systolic dysfunction, potentially guiding initial management decisions. FS, although somewhat less accurate, can still provide complementary information when used alongside EPSS.

### Limitations

This study has several limitations that should be acknowledged. First, it was conducted at a single center with a relatively small sample size, which may limit the generalizability of the findings. Second, the design was observational and did not include long-term follow-up, potentially underestimating delayed outcomes such as rehospitalization or late mortality. Third, echocardiographic measurements in the ED were performed by emergency physicians, which may introduce variability compared with cardiology specialists. However, this limitation also reflects real-world practice, where bedside assessments are often performed by non-cardiologists. Finally, although we compared EPSS and FS with both EF and GLS, strain imaging was performed only with vendor-specific software and not validated across multiple platforms, which may affect reproducibility in other settings.

## 5. Conclusions

In conclusion, this study demonstrates that both EPSS and FS, when measured at the bedside in the ED, strongly correlate with left ventricular systolic function as assessed by EF and GLS. EPSS, with its superior diagnostic performance and closer alignment with GLS, appears to be a particularly valuable surrogate for advanced strain imaging in the acute care setting. FS, while somewhat less accurate and more dependent on ventricular geometry and loading conditions, nonetheless remains a practical and usable parameter when EPSS measurement is limited. These findings support the integration of simple echocardiographic indices such as EPSS and FS into the routine evaluation of dyspneic patients, especially in resource-constrained or time-sensitive clinical environments.

## Figures and Tables

**Table 1:** Baseline demographics, vital signs, and comorbidities.

Variable	Value
Age, year	61.7 ± 14.2; median 64 (range 21–85)
Male sex, *n* (%)	38 (60.3)
**Vital Signs**	**Mean ± SD**
Systolic Blood Pressure (mmHg)	142.5 ± 27.5
Diastolic Blood Pressure (mmHg)	81.3 ± 15.0
Heart rate (beats/min)	92.5 ± 14.9
Oxygen saturation (%)	92.9 ± 5.7
Temperature (°C)	36.32 ± 0.41
Respiratory rate (breaths/min)	18.7 ± 4.1
**Comorbidities**	***n*** **(%)**
Any comorbidity	56 (88.9)
Hypertension	34 (54.0)
Coronary artery disease	25 (39.7)
Diabetes mellitus	25 (39.7)
Heart failure	15 (23.8)
Chronic Obstructive Pulmonary Disease	6 (9.5)
Chronic kidney disease	6 (9.5)
Malignancy	1 (1.6)
Other	10 (15.9)

**Table 2:** Final diagnoses and emergency department outcomes.

Final Diagnosis	*n* (%)
Acute Coronary Syndrome	22 (34.9)
Decompensated heart failure	17 (27.0)
Pneumonia	8 (12.7)
Nonspecific	10 (15.9)
Pulmonary Embolism	2 (3.2)
Myopericarditis	2 (3.2)
Cardiomyopathy	1 (1.6)
Acute Renal Deficiency	1 (1.6)
**Emergency department outcomes**	
Intensive Care Unite admission	34 (54.0)
Ward admission	9 (14.3)
Discharge	20 (31.7)

**Table 3:** Descriptive statistics of echocardiographic measurements.

Variables	*n*	Mean	SD	Median	Range
GLS (%)	63	14.28	3.87	14.0	5.3–24.3
Biplane EF (%)	63	46.58	11.81	48.1	22.3–63.3
Fractional Shortening (%)	63	26.54	8.18	28.0	9.4–45.0
EPSS (mm)	63	8.7	3.5	8.3	3.0–19.0

**Table 4:** Summary of ROC analyses.

Endpoint	Test	AUC	Optimal Threshold (Youden)	Sensitivity	Specificity	Youden J
Reduced EF	EPSS (mm)	0.922	≥7.05	0.861	0.815	0.676
Reduced EF	FS (%)	0.874	≤28.15	0.778	0.815	0.593
Reduced GLS	EPSS (mm)	0.949	≥7.05	0.868	0.880	0.748
Reduced GLS	FS (%)	0.865	≤24.95	0.684	0.920	0.604

**Table 5:** Spearman correlation coefficients between echocardiographic parameters.

Variables	GLS	Biplane EF	FS	EPSS
**GLS**	1	0.866 (*p* < 0.001)	0.714 (*p* < 0.001)	−0.782 (*p* < 0.001)
**Biplane EF**	0.866 (*p* < 0.001)	1	0.773 (*p* < 0.001)	−0.834 (*p* < 0.001)
**FS**	0.714 (*p* < 0.001)	0.773 (*p* < 0.001)	1	−0.784 (*p* < 0.001)
**EPSS**	−0.782 (*p* < 0.001)	−0.834 (*p* < 0.001)	−0.784 (*p* < 0.001)	1

**Table 6:** Kappa agreement analyses between emergency FS/EPSS classifications and reference echocardiographic parameters.

Test (Cut-Off)	Reference	Sens	Spes	PPV	NPV	Kappa (SE), *p*
EPSS ≥ 7.0 mm	Reduced EF	0.861	0.815	0.861	0.815	0.676 (0.094), <0.001
EPSS ≥ 7.0 mm	Reduced GLS	0.868	0.880	0.917	0.815	0.738 (0.086), <0.001
FS ≤ 25%	Reduced EF	0.694	0.889	0.893	0.686	0.563 (0.100), <0.001
FS ≤ 25%	Reduced GLS	0.684	0.920	0.929	0.657	0.566 (0.097), <0.001

EPSS ≥ 7.0 mm: EF: 31/5/5/22, Strain: 33/3/5/22; FS ≤ 25%: EF: 25/3/11/24, Strain: 26/2/12/23 (TP/FP/FN/TN).

## Data Availability

The original contributions presented in this study are included in the article. Further inquiries can be directed to the corresponding author.
